# Lost in translation: The 3'-UTR of *IGF1R* as an ancient long noncoding RNA

**DOI:** 10.1093/emph/eoy008

**Published:** 2018-03-16

**Authors:** Avantika Mainieri, David Haig

**Affiliations:** Department of Organismic and Evolutionary Biology, Harvard University, 26 Oxford Street, Cambridge, MA 02138, USA

**Keywords:** microRNA, IGF1R, H19, genomic imprinting, noncoding RNA, evolution

## Abstract

**Background and objectives:**

The insulin-like growth factor (IGF) signaling system is a major arena of intragenomic conflict over embryonic growth between imprinted genes of maternal and paternal origin and the IGF type 1 receptor (IGF1R) promotes proliferation of many human cancers. The 3'-untranslated region (3'-UTR) of the mouse *Igf1r* mRNA is targeted by miR-675-3p derived from the imprinted *H19* long noncoding RNA. We undertook a comparative sequence analysis of vertebrate *IGF1R* 3'-UTRs to determine the evolutionary history of miR-675 target sequences and to identify conserved features that are likely to be involved in post-transcriptional regulation of IGF1R translation.

**Methodology:**

Sequences of *IGF1R* 3'-UTRs were obtained from public databases and analyzed using publicly available algorithms.

**Results:**

A very long 3'-UTR is a conserved feature of vertebrate *IGF1R* mRNAs. We found that some ancient microRNAs, such as let-7 and mir-182, have predicted binding sites that are conserved between cartilaginous fish and mammals. One very conserved region is targeted by multiple, maternally expressed imprinted microRNAs that appear to have evolved more recently than the targeted sequences.

**Conclusions and implications:**

The conserved structures we identify in the *IGF1R* 3'-UTR are strong candidates for regulating cell proliferation during development and carcinogenesis. These conserved structures are now targeted by multiple imprinted microRNAs. These observations emphasize the central importance of IGF signaling pathways in the mediation of intragenomic conflicts over embryonic growth and identify possible targets for therapeutic interventions in cancer.

## BACKGROUND AND OBJECTIVES

Parent-specific gene expression (genomic imprinting) is proposed to be an outcome of evolutionary conflict between genes of maternal and paternal origin [1]. Soon after the first presentation of this hypothesis, three imprinted loci were identified in mice: *Igf2* was a paternally expressed gene (PEG) that encoded insulin-like growth factor II (IGF-II) [2]; *Igf2r* was a maternally expressed gene (MEG) that encoded a receptor for IGF-II [3]; and *H19* was a MEG that encoded a long noncoding RNA [4, 5]. *Igf2* promoted fetal growth whereas *Igf2r* inhibited fetal growth [6]. These phenotypic effects were broadly consistent with the theoretical prediction that PEGs should enhance prenatal growth and MEGs inhibit prenatal growth [7] with unimprinted genes favoring an intermediate level of growth [8].

*H19* and *Igf2* were each other’s closest chromosomal neighbors and shared common regulatory elements [9, 10]. Therefore, the intuitive prediction was that *H19* (a MEG) should act antagonistically to *Igf2* (a PEG) and function as a fetal growth inhibitor. However, despite more than two decades of study, the functions of *H19* remain poorly understood. Comparisons among *H19* genes from several eutherian species identified conserved secondary structures [11]. One conserved hairpin was shown to be processed as the pre-miRNA for miR-675 [12]. The 3'-untranslated region (3'-UTR) of the murine *Insulin-like growth factor 1 receptor* (*Igf1r*) gene was subsequently found to possess two target sites for miR-675-3p [20]. IGF1R mediates the fetal growth-promoting effects of IGF-II [6] and binding of miR-675-3p to the 3'-UTR of *Igf1r* mRNA inhibits translation of the receptor [13]. Therefore, miR-675-3p is predicted to function as a maternally expressed inhibitor of fetal growth.

The initial impetus for the present study was to examine the evolutionary history of the miR-675-3p target sequences reported for mouse *Igf1r* mRNA [13]. We soon found that one of these targets was absent from the *IGF1R* genes of rats and humans, but the second target was conserved in the *IGF1R* gene of a cartilaginous fish (*Callorhinchus milii*) where it was located in one of the most highly conserved regions of the entire 7-kb 3'-UTR. This discovery prompted us to undertake an evolutionary analysis of the full length of the *IGF1R* 3'-UTR to complement an earlier evolutionary analysis of the *IGF1R* coding sequence [14].

Our principal objectives were 2-fold. First, we wished to understand better the evolution of genomic imprinting in mammalian development and the interplay between PEGs, MEGs and unimprinted genes in the IGF signaling system. Second, we wished to contribute to an understanding of the control of IGF1R expression because of the receptor’s important role in cancer biology. IGF1R mediates growth-promoting effects of both IGF-I and IGF-II [6] and is highly expressed in many cancers. Furthermore, cells with inactivated *IGF1R* are resistant to oncogenic transformation [15]. For these reasons, IGF1R was promoted as the Achilles’ heel of most, if not all, cancers [16]. Such high hopes were dashed by disappointing results of clinical trials of therapies targeting IGF1R [17, 18]. These expensive failures may partly reflect imperfect understanding of the regulation of *IGF1R* expression. Highly conserved structures within the 3'-UTR deserve consideration as targets for more effective therapeutic interventions.

## METHODOLOGY

Sequences were aligned using BLAST with default settings. miRanda [19], TargetScan [20] and reports in the literature were used to identify putative target sequences of microRNAs. Polyadenylation sites of human *IGF1R* mRNAs were identified in APASdb [21] and APAdb [22] databases. Potential secondary structures of *IGF1R* 3'-UTRs were explored using mFold [23].

## RESULTS

The most abundant human *IGF1R* transcript exceeds 12 kb in length of which 1 kb is 5'-UTR, 4 kb is coding sequence and 7 kb is 3'-UTR. We will call this the ‘long transcript’. A number of shorter transcripts with alternative polyadenylation sites are reported in APASdb and APAdb databases. The second most common transcript in these databases is 6.4 kb in length with a 1.3 kb 3'-UTR. We will call this the ‘short transcript’. The short transcript possesses an atypical polyadenylation site (upstream sequences do not contain canonical polyadenylation signals).

Putative 3'-UTRs of the long transcript were obtained from genomic sequences of *Homo sapiens* (human), *Monodelphis domesticus* (opossum), *Pelodiscus sinensis* (turtle) and *C. milii* (ghostshark). The stop codon of *IGF1R* mRNAs was easy to identify. Significant similarity was also apparent between the 3' end of a human *IGF1R* cDNA (NM_000875.4) and sequences from the other species (Fig. 2a). All nucleotides from the end of the stop codon to the end of UCUGUAUGCA were considered to constitute 3'-UTRs of lengths 7087 (*Homo*), 7517 (*Monodelphis*), 7135 (*Pelodiscus*) and 9136 (*Callorhinchus*) nucleotides.

**Figure 1.**
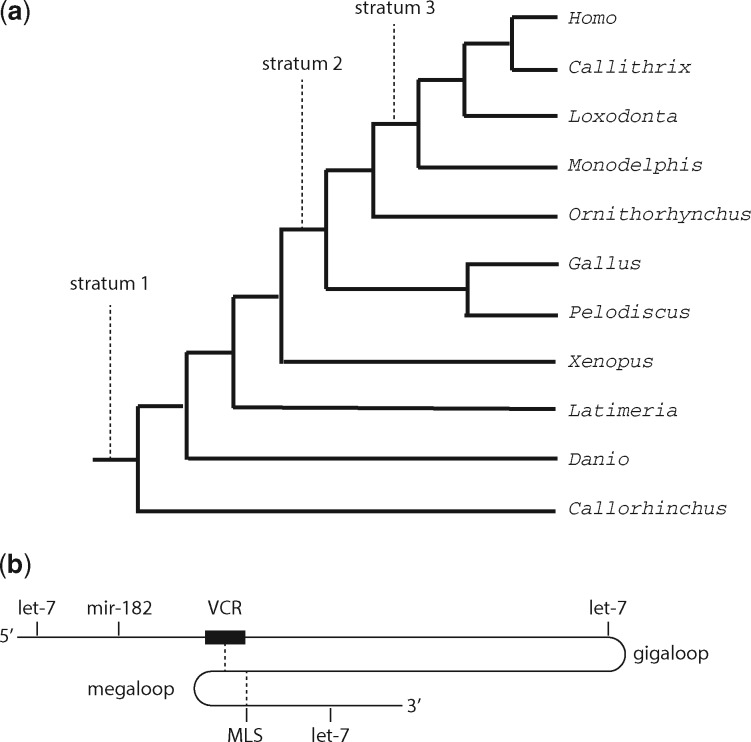
(**a**) Phylogenetic relationships of species whose *IGF1R* 3'-UTRs are used in this study. The origin of genomic imprinting and the *H19* long noncoding RNA is thought to coincide with stratum 3 (in an ancestor of marsupial and eutherian mammals). (**b**) Landmarks on the 7-kb 3'-UTR of the human ‘long’ *IGF1R* mRNA including conserved let-7-5p and miR-182 target sites. The 1.3 kb 3'-UTR of the ‘short’ transcript terminates within the VCR, which also includes a conserved miR-675-3p-binding site. The 0.8-kb megaloop is formed by pairing of the megaloop stems (MLS). The 4.8-kb gigaloop is a putative structure formed by pairing of VCR and a complementary sequence (cVCR)

Comparisons among the four focal sequences allowed ‘excavation’ of the deep history of the *IGF1R* 3'-UTR. Features shared by *Homo* and *Callorhinchus* can be inferred to have been present in the last common ancestor of extant jawed vertebrates (stratum 1); features shared by *Homo* and *Pelodiscus* to have been present in the last common ancestor of extant amniotes (stratum 2); and features shared by *Homo* and *Monodelphis* to have been present in the last common ancestor of therian mammals (stratum 3). Stratum 3 also corresponds to the conjectured origin of genomic imprinting and microRNA-675 (Fig. 1a). If a feature is not shared between two sequences, it may have been present in the ancestor but lost in one of the descendent lineages or have been absent in the ancestor but gained in one of the descendent lineages. Data from *IGF1R* 3'-UTRs of other species will be reported where these provide more precise timing for particular evolutionary events.

**Figure 2.**
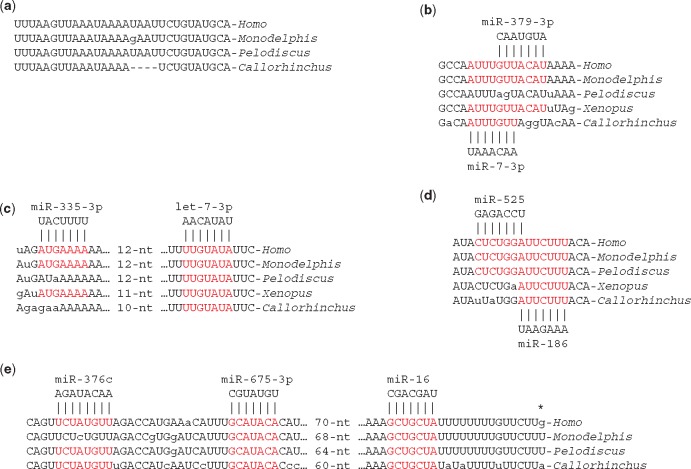
Conserved sequences of stratum 1 (shared by *Homo* and *Callorhinchus IGF1R* 3'-UTRs): (**a**) the 3' end of the long *IGF1R* transcript; (**b**) a miR-7-3p target site that has been lost from the *Pelodiscus* sequence; (**c**) let-7-3p target site; (**d**) miR-186 target site; (**e**) The VCR with predicted binding sites for miR-376c, miR-675 (derived from the imprinted *H19* RNA) and miR-16. The location of the start of the poly(A) tail of the ‘short’ (6.4 kb) human *IGF1R* mRNA is marked with an asterisk

In comparisons of *Homo* and *Callorhinchus* 3'-UTRs, BLAST detected a few small islands of conservation in a sea of otherwise unalignable sequence. The roughly 10% of alignable sequence constitutes stratum 1. By contrast, BLAST aligned the *Homo* and *Pelodiscus* sequences for roughly half their length (stratum 2) and the *Homo* and *Monodelphis* sequences for most of their length (stratum 3). Our analysis will focus on the deeply conserved structures of strata 1 and 2. Figure 1b provides landmarks for orientation in the following discussions of the *IGF1R* 3'-UTR.

### Stratum 1

Stratum 1 consists of features shared by *Homo* and *Callorhinchus* 3'-UTRs. BLAST aligned two segments longer than 100 nucleotides. The longest alignment was a stretch of 300 nucleotides (78% identity) at the 3' end of the long (12 kb) human *IGF1R* mRNA. In addition to the polyadenylation site, this region contains a miR-7-3p target site that has been lost from the *Pelodiscus* sequence (Fig. 2b), a conserved let-7-3p target site (Fig. 2c) and a conserved miR-186 target site (Fig. 2d). All these sites were predicted for the human 3'-UTR by miRanda.

The second longest alignment was a stretch of 175 nucleotides (75% identity) a kilobase from the start of the 3'-UTR that we will call the ‘very conserved region’ (VCR; Fig. 2e). The VCR includes the polyadenylation site of the short (6.4 kb) human *IGF1R* mRNA. The VCR contains a target site for miR-675 [13], a target site for the miR-16 family of microRNAs [24, 25] and a target site for miR-376c [26]. It is notable that miR-675 and miR-376 are both imprinted and maternally expressed [12, 27]. The miR-675 and miR-16 target sites are proximal to the polyadenylation site and thus included within the short transcript. The miR-376 binding site occurs distal to the end of the short transcript.

**Figure 3.**
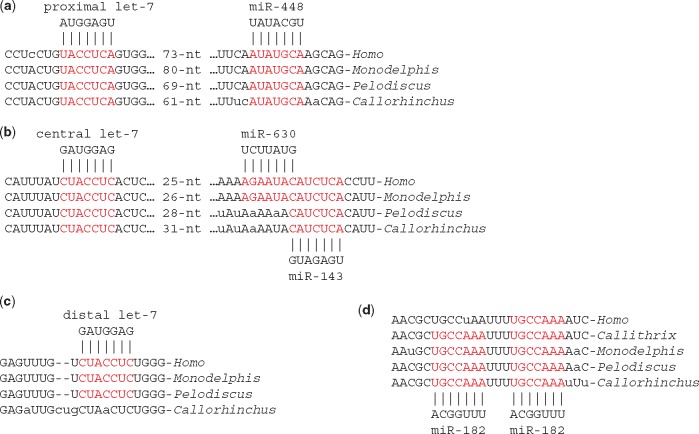
Conserved microRNA target sequences of stratum 1. (**a**–**c**) *let-7* target sequences; (**d**) tandem target sequences for miR-182. microRNAs are represented by their seed sequence. Nucleotides of candidate target sequences are shown in red. Nucleotides that differ from the consensus in lower case

Shorter alignments included other predicted target sequences for microRNAs. The 3'-UTR of human *IGF1R* possesses three target sequences for *let-7*-5p microRNAs [28]. The proximal and central target sequences are conserved in the ghostshark sequence whereas the distal target sequence is recognizable, but imperfect, in the ghostshark sequence (Fig. 3a–c). The proximal *let-7*-5p target sequence is close to a conserved miR-448 target sequence and the central *let-7*-5p target sequence close to a conserved miR-143 target sequence (Fig. 3a–b) [29, 30]. Another short region of conservation contains tandem target sequences for miR-182 that are present in *Callorhinchus*, *Pelodiscus*, *Monodelphis* and *Callithrix* (marmoset) *IGF1R* genes. Only one of these tandem targets is conserved in the human *IGF1R* gene (Fig. 3d) [31].

Sequence conservation over such large evolutionary distances suggests that the conserved sequences are deeply constrained by function. Postranscriptional regulation of *IGF1R* by let-7, miR-7 and miR-182 are plausible candidates for such ancient functions because these microRNA families are themselves ancient, conserved between protostomes and deuterostomes [32–34]. By contrast, miR-675 is known only from marsupial and eutherian mammals [35, 36] and miR-376 only from eutherian mammals [27]. Either the miR-675 and miR-376 binding sites were targets for unidentified ancient microRNAs, perhaps still present in *Pelodiscus* and *Callorhinchus*, or the more recent imprinted microRNAs evolved to target sequences that were conserved for functions unrelated to binding by microRNAs. If the latter scenario is correct, then the VCR is likely to have been an original target of miR-675 and miR-376 which evolved to target its sequence. By contrast to the deeply conserved target site for miR-675 in the VCR, the second reported target site for miR-675 [13] is found only in the *Igf1r* 3'-UTRs of house mice and their close relatives.

The two longest regions of sequence conservation between ghostshark and human *IGF1R* in our study corresponded to the 3' ends of the human ‘short’ (6.4 kb) and ‘long’ (12 kb) *IGF1R* transcripts. This hints that alternative polyadenylation at these sites is anciently conserved. Other short regions of conservation between *Homo* and *Callorhinchus* sequences corresponded to polyadenylation sites of less common human *IGF1R* transcripts in APASdb and APAdb (data not shown). An earlier comparison of the 3'-UTRs of eight human genes with orthologous sequences in *Squalus acanthias*, another cartilaginous fish detected conservation of the 3' end of the 3'-UTR for six of eight genes [37].

### Stratum 2

Stratum 2 consists of features shared by *Homo* and *Pelodiscus IGF1R* 3’-UTRs. The strongest conservation was a 388-nt alignment that includes the VCR (85% identity with 3% gaps) and a 497-nt alignment at the 3' terminus (82% identity with 8% gaps). This degree of conservation is similar, if not greater, than found for the *IGF1R* coding sequence (79% identity with 1% gap).

**Figure 4.**
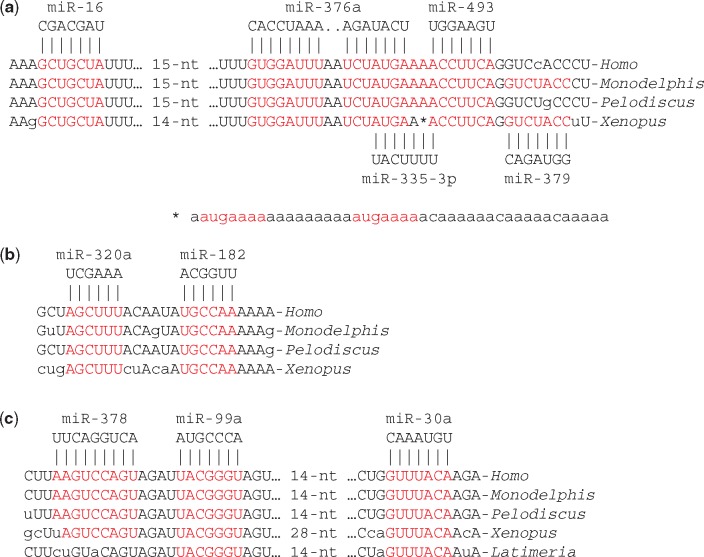
The ‘extended VCR’ of stratum 2 (shared by *Homo* and *Pelodiscus* sequences): (**a**) miR-16 target site (also shown in Fig. 2e) and nearby target sites for miR-376a, miR-335-3p, miR-493 and miR-379 (the *Xenopus* sequence contains a 44-bp insertion at the site of the asterisk that includes two target sites for miR-335-3p are shown in red); (**b**) conserved pair of target sites for miR-320a and miR-182; (**c**) conserved triplet of target sites for miR-378, miR-99a and miR-30a

Although the facile alignment of *Homo* and *Callorhinchus* 3’-UTRs ends shortly after the miR-16 binding site of the VCR at the location of the polyadenylation site for the short human transcript (Fig. 2e), strong similarity continues beyond this point for *Homo*, *Monodelphis*, *Pelodiscus* and *Xenopus* sequences. This ‘extended VCR’ contains a remarkable concentration of predicted binding sites for microRNAs, including miR-335-3p [38], miR-376a [26] and miR-493 [39]. The *Monodelphis* and *Xenopus* sequences also have a predicted binding site for miR-379-5p adjacent to the miR-493 binding site (Fig. 4a). miR-376a, miR-379 and miR-493 are encoded in a large cluster of maternally expressed imprinted microRNAs found only in eutherian mammals [27]. miR-335 is a paternally expressed imprinted microRNA located in the second intron of its *MEST* host gene [40, 41]. miR-335 is well conserved in armadillo and elephant *MEST* genes but absent from the *MEST* genes of opossum and platypus. Therefore, conservation of the extended VCR appears to be more ancient than the imprinted miRNAs that currently target its sequence. The simplest interpretation is that the extended VCR has important functions independent of binding of imprinted microRNAs and that these microRNAs evolved to target the conserved sequence.

**Figure 5.**
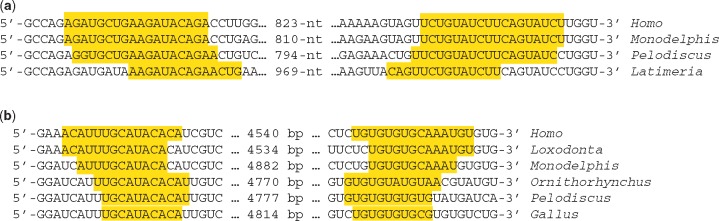
Nucleotides predicted to form the stems of (**a**) megaloop and (**b**) gigaloop are shaded

A notable feature of stratum 2 is a pair of complementary sequences, 800 nucleotides apart, that are predicted to form the stems of a strong double helix (18 bp, –32.3 kcal/mol). Similar complementary sequences are found in *Xenopus* and *Latimeria* (coelacanth) 3'-UTRs (Fig. 5a), but neither in *Danio* (zebrafish) nor *Callorhinchus* 3'-UTRs. The region between the 5' and 3' stems will be called the ‘megaloop’. The megaloop contains, among other features, a conserved pair of target sites for miR-320a and miR-182 (Fig. 4b) and a conserved triplet of target sites for miR-378, miR-99a and miR-30a (Fig. 4c). All of these features are also found in the 3'-UTR of *Xenopus silurana* and are therefore predicted to have been present in the ancestor of all extant tetrapods. The miR-99a and miR-30a target sites are found in the *Latimeria* sequence and can therefore be inferred to have been present in the last common ancestor of tetrapods and lobe-finned fishes.

### Alternative secondary structures

*IGF1R* mRNAs were longer than the maximum size that could be submitted to mFold. Therefore, we explored folding of the 7-kb 3’-UTR of the long human transcript without the 5'-UTR and coding sequence. The mFold algorithm makes many simplifying assumptions and the predicted structures of such a long transcript are unlikely to be accurate in all details. For example, the algorithm does not consider the possibility of pseudo-knots, triplexes, G-quadruplexes and other complex structures, nor does it take account of interactions between the RNA sequence and RNA-binding proteins. Our purpose in this exploratory analysis was to search the predicted structures for repeated stable features involving sequences that were already of interest from our evolutionary analysis.

The 5' and 3' stems of the megaloop (see above) formed a strong double helix in many secondary structures predicted by mFold. By contrast, the miR-675 binding site of the VCR was often located within a long single-stranded bulge or in weakly bonded secondary structures. Thus, the VCR exhibits the intriguing combination of highly conserved primary sequence (suggesting important conserved function) and lack of clear secondary structure. Similarly, regions of high sequence conservation between human and mouse 3'-UTRs of the *MeCP2* gene do not form stable secondary structures [42]. We suggest that the VCR is analogous to ‘intrinsically disordered’ domains of proteins that have important regulatory functions [43]. A flat energy landscape in the vicinity of the VCR means that this region is intrinsically mobile and potentially able to interact with proteins, microRNAs and other regions within the *IGF1R* 3'-UTR.

Although the VCR was ‘disordered’ in many predicted structures, the miR-675 target site was sometimes recovered as part of a strong double helix (15 bp, –25.8 kcal/mol) formed with a complementary sequence (cVCR), 4.5 kb distant located between the stems of the megaloop (Fig. 5b). We will refer to this putative pairing of VCR and cVCR as the gigaloop. From the temporal perspective of transcription, the 5'-stem of the gigaloop (VCR) is transcribed first, then the 5'-stem of the megaloop, then the 3'-stem of the gigaloop (cVCR), then the 3'-stem of the megaloop (Fig. 1b). Therefore, the stems of the gigaloop have an opportunity to pair before the 3'-stem of the megaloop is transcribed. One intriguing scenario is that the VCR initially adopts an inactive conformation paired to the cVCR but acquires its active, open conformation by formation of the megaloop. We are unable to tell whether the megaloop and gigaloop could both exist in the same folded structure because pseudo-knots (part of a loop forming a stem with another sequence outside of its loop) are precluded by the mFold algorithm.

The age of the VCR-cVCR double helix is unclear. A strong double helix is predicted for the 3'-UTRs of *Loxodonta* (elephant, 13 bp, –21.5 kcal/mol) and *Monodelphis* (opossum, 12 bp, –19.4 kcal/mol), but the cVCR is poorly conserved in *Ornithorhynchus*, *Pelodiscus* and *Gallus*. The homologous sequences of the latter species consist mostly of simple UG repeats, but when the VCR and repetitive sequences are induced to pair, mFold predicts a 12 bp double helix in *Ornithorhynchus* (–18.9 kcal/mol), a 11 bp double helix in *Pelodiscus* (–16.7 kcal/mol) and a 10 bp double helix in *Gallus* (–17.9 kcal/mol). Because of the simplicity of the UG repeats and the general ‘stickiness’ of Us and Gs (G and U can form ‘wobble’ pairs with each other as well as the standard Watson-Crick pairing of G with C and U with A), we consider the evidence for functional pairing of VCR and cVCR to be equivocal in *Pelodiscus* and *Gallus*. A sequence complementary to the VCR within the megaloop was found in neither the *Xenopus* nor *Latimeria* sequences.

### 3'-UTRs as Boolean devices

Individual microRNAs (in complex with Argonaute proteins) have been reported to both inhibit and promote translation of mRNAs [44, 45]. The assemblage of microRNAs within a cell can be considered a ‘news report’ of cellular conditions that is ‘read’ by the 3'-UTRs of genes that have ‘subscriptions’ (target sites) to the news service. As a result of adaptive natural selection, each 3'-UTR is expected to respond adaptively to some microRNAs but not others. A notable feature of our comparative analysis is the clustering of microRNA-binding sites (Figs 1–4). Close proximity of binding sites could facilitate antagonistic or synergistic interactions among Argonaute–microRNA complexes (for example by steric hindrance). This could enable the 3'-UTR to perform the equivalent of simple logical calculations. If an effect Z was achieved by the binding of both miR-X and miR-Y, then the interaction of the microRNAs would be formally equivalent to the Boolean operation X AND Y = Z. If instead, miR-X and miR-Y had redundant effects, this would be equivalent to X OR Y = Z. If binding of miR-Y prevented miR-X causing Z, this would be equivalent to X NOT Y = Z. In a similar manner, combinations of riboswitches have been proposed to perform Boolean computations [46, 47].

The long 3'-UTR of *IGF1R* (> 7000 nucleotides) may function as a ‘computational device’ that responds to information about cellular conditions provided by the binding of miRNAs (among other factors). miRDB lists 185 microRNAs binding human *IGF1R* 3'-UTR, many of them with multiple target sites [48]. A comparison to other long transcripts with predicted target sites for miR-675-3p (*KCNN3*, *TAOK1*, *PYGO1*, *MRPL19*, *ANKH* and *ADAM22*) suggests that 185 is a ‘middling’ number of predicted microRNAs for a transcript of this length. Even if many of these predicted sites are nonfunctional, the potential for allosteric interactions within and among clusters of binding sites could enable the 3'-UTR to perform complex, hierarchically organized computations to determine if and when the mRNA is translated. Most research on the effects of microRNAs on expression of *IGF1R* investigates effects of individual microRNAs on protein or mRNA levels. If the ‘computational model’ of 3'-UTR function is correct, the investigation of one microRNA at a time may give inconsistent results because the meaning of a microRNA ‘word’ must be understood in the context of the cellular ‘sentence’ of co-occurring microRNAs.

### Imprinted microRNAs and the regulation of *IGF1R*

Our comparative analysis of the *IGF1R* 3'-UTR has uncovered deeply conserved sequences that are targeted by multiple maternally expressed imprinted microRNAs. An evolutionary interpretation of this finding requires a digression about the co-evolution of microRNA seeds and their mRNA targets. A typical microRNA binds to the 3'-UTRs of many genes [49]. Therefore, mutations to the seed sequence of ancient microRNAs are strongly selected against because each mutation affects the expression of many genes. By contrast, a change to an mRNA’s target sequence directly affects the expression of only that gene. For this reason, the mRNAs that a microRNA targets are more evolutionarily labile than the seed sequence that recognizes these targets [50].

Additional evolutionary complexities arise when an imprinted microRNA targets an unimprinted mRNA because mutations in the seed sequence are subject to selection only when the microRNA is inherited from parents of one sex but mutations in the target sequence are subject to selection when inherited from parents of both sexes. Therefore, changes that promote the fitness of the targeted sequence need not promote the fitness of the targeting sequence. For example, PEGs are predicted to favor more fetal growth than unimprinted genes (and MEGs favor less fetal growth). A novel maternally expressed microRNA that targeted the biallelically expressed *IGF1R* 3'-UTR would be favored by natural selection if it inhibited expression of the protein because signaling via IGF1R promotes fetal growth. Such targeting would, in turn, favor mutations of the target site that eliminated binding by the microRNA. For this reason, many interactions between imprinted microRNAs and unimprinted targets are expected to be evolutionarily short-lived. But the simple expedient of eliminating the target is foreclosed if the targeted sequence performs other essential functions.

These considerations suggest an evolutionary model for post-transcriptional regulation of unimprinted mRNAs by imprinted microRNAs. Maternally expressed microRNAs target conserved regions of the *IGF1R* 3'-UTR both because these regions are functionally important and because natural selection favors maintenance of the target sequence for reasons unconnected to targeting by the microRNAs. By this process, multiple imprinted microRNAs have come to target the highly conserved sequences of the extended VCR. Our analysis suggests that the VCR may have been the original functional target of the imprinted microRNAs because the targeted sequences are evolutionarily older than the imprinted microRNAs that bind them (the microRNA seed sequences evolved to match the target sequences rather than the other way round).

Because of the distinct selective forces acting on imprinted and unimprinted loci, the unimprinted *IGF1R* 3'-UTR is predicted to undergo evolutionary change at other sites to compensate for inhibitory effects of imprinted microRNAs. Such changes are predicted to stabilize the relationship between the 3'-UTR and maternally expressed microRNAs because mutations to a microRNA that eliminated its interaction with *IGF1R* would be selected against because these would increase expression of IGF1R and increase fetal growth. Once the seed sequence of an imprinted microRNA is ‘evolutionarily tethered’ in this manner, other unimprinted mRNAs will evolve binding sites for the microRNA if such binding enhances the fitness of the genomic sequence encoding the unimprinted mRNA. As a result, some of the interactions between an imprinted microRNA (or long noncoding RNA) and unimprinted RNAs will promote the fitness of the ‘targeting’ sequence and others the fitness of the ‘targeted’ sequences. These complexities may partly explain the difficulty of assigning an unambiguous function to *H19* and miR-675 in the regulation of cell proliferation. *H19* inhibits cell proliferation in some systems [51, 52] but promotes proliferation in other systems [53, 54] and has been interpreted as both a tumor suppressor [55, 56] and oncogene [57].

IGF1R protein mediates the growth-promoting effects of IGF2. Therefore, evolutionary theory would predict *IGF1R* to be paternally expressed if it were imprinted. However, inactivating mutations of *Igf1r* in mice provide no evidence of imprinting [58] and effects of *IGF1R* mutations on human growth appear independent of parental origin [59]. The absence of imprinting of *IGF1R*, despite its role in promoting fetal growth, could be explained if the gene’s effects are not dosage sensitive, because selection for imprinted expression is weak or nonexistent if one active copy is functionally equivalent to two [60]. Data on haplosufficiency versus haploinsufficiency of effects of *IGF1R* on prenatal growth are ambiguous and differ between mice and humans. Mice with one inactivated copy of *Igf1r* do not exhibit growth deficits at birth or before weaning [58, 61]. In one of these knockouts, inactivation of one copy of *Igf1r* did not reduce steady-state levels of mRNA [58] whereas, in another knockout, inactivation of one allele was associated with a 50% reduction in the cell-surface expression of Igf1r protein [61]. On the other hand, some children with heterozygous mutations of *IGF1R* exhibit intrauterine growth retardation although this phenotype has variable penetrance even for children with the same mutation [62].

Although *IGF1R* transcripts are not known to exhibit parent-specific expression, the *IGF1R* genomic locus contains a sequence within intron 2 that is more heavily methylated when inherited from mothers than from fathers [63]. Moreover, the first intron of human *IGF1R* contains the promoter of a long noncoding RNA *IRAIN* that is antisense to the 5'-UTR of *IGF1R*, interacts with the *IGF1R* promoter [64], and is exclusively transcribed from the paternally derived allele [65]. If *IRAIN* acts in *cis*, then a functional interaction with the *IGF1R* promoter could result in differential expression of the maternal and paternal alleles of *IGF1R*. Clearly, there are complexities in the regulation of *IGF1R* expression yet to be explicated.

## CONCLUSIONS AND IMPLICATIONS

Evolutionary sequence analysis is not a substitute for experimental investigation of mechanisms but evolutionary analysis can guide experiment by identifying structures of interest. The 7 kb 3'-UTR of the major *IGF1R* transcript compares to a median length of human 3'-UTRs of about 1.2 kb [66]. Our analysis suggests that a very long 3'-UTR is an ancient feature of *IGF1R* genes and that the 3'-UTR contains deeply conserved sequences. The sequence we call the VCR stands out as deserving detailed investigation for three reasons. First, its primary sequence is deeply conserved and must therefore be strongly constrained by function. Second, the VCR is located immediately adjacent to the polyadenylation site of the short (6.4 kb) human *IGF1R* mRNA. Third, the VCR is targeted by multiple imprinted microRNAs (including miR-675-3p derived from the *H19* RNA). IGF signaling has been a focus of evolutionary conflict between genes of maternal and paternal origin over relative proliferation of particular cell types as witnessed by the opposite imprinting of *IGF2* and *IGF2R* [67, 68]. For this reason, regions of the *IGF1R* mRNA targeted by imprinted microRNAs are strong candidates for effects on the control of cell proliferation.

3'-UTRs play many important roles in the control of protein translation and function [69]. The 3'-UTRs of *IGF1R* mRNAs have been little studied, beyond the identification of microRNA target sites, perhaps because of the transcripts’ length and complexity. Remarkably, we have found no articles that discuss polyadenylation of mammalian *IGF1R* mRNAs, even though polyadenylation is considered a requirement for efficient translation. The only papers we found on *IGF1R* polyadenylation came from teleost fish. One study in turbot found that a 13-kb *IGF1R* transcript was polyadenylated in oocytes and early embryos but not in larvae or adult somatic tissues [70]. Our study found that sequences near the polyadenylation sites of the human 6.4 and 12 kb mRNAs are conserved between mammals and cartilaginous fish (and other small regions of ancient conservation included additional polyadenylation sites). The polyadenylation of human *IGF1R* transcripts and the dynamics of alternative polyadenylation deserve future study.

Our study investigated the 3'-UTR of a single gene (*IGF1R*) with a focus on its interaction with a single imprinted microRNA (miR-675-3p). We have undoubtedly explored only the tip of the iceberg in understanding the role of noncoding RNAs in intragenomic conflicts. The largest clusters of microRNAs in the human and mouse genomes are imprinted. These include a cluster of maternally expressed microRNAs (C14MC) present in all eutherian mammals [27] and the paternally expressed C19MC cluster of primates [71, 72] and *Sfmbt2* cluster of muroid rodents [73]. The maternally expressed *H19* long noncoding RNA is not only the substrate for the production of miR-675 but also acts as a competing endogenous RNA (ceRNA) or ‘molecular sponge’ for absorbing other microRNAs [74, 75]. Similarly, *maternally expressed gene 3* (*MEG3*), a close genomic neighbor of the C14MC microRNAs, is an imprinted long noncoding RNA with ceRNA functions [76]. Conflict breeds complexity. Untangling the web of interactions is a task for future experimental and evolutionary studies.

**Conflict of interest:** None declared.
